# Motivation and Commitment to Sports Practice During the Lockdown Caused by Covid-19

**DOI:** 10.3389/fpsyg.2020.622595

**Published:** 2021-01-11

**Authors:** Marta Leyton-Román, Ricardo de la Vega, Ruth Jiménez-Castuera

**Affiliations:** ^1^Sport of Studies Center, Rey Juan Carlos University, Madrid, Spain; ^2^Departament of Physical Education, Sport and Human Movement, Faculty of Teacher Training and Education, Autonomous University of Madrid, Madrid, Spain; ^3^Didactic and Behavioural Analysis in Sport Research Group, Faculty of Sport Sciences, University of Extremadura, Cáceres, Spain

**Keywords:** motivation, commitment, sports, COVID-19, self-determination theory

## Abstract

In Spain, the state of alarm declared on March 14, 2020 caused changes in the population in relation to the habits of physical activity and sports practice. This study analyzed what motivational variables predicted the self-efficacy and commitment to sports practice, as well as the differences according to gender, during lockdown and the progressive de-escalation caused by COVID-19, using the theory of self-determination as a theoretical framework. The study sample was conformed of 179 subjects (90 men and 89 women) between 18 and 65 years of age (*M* = 28.64; *SD* = 10.28). The Behavioral Regulation in Sport Questionnaire (BRSQ), the Psychological Need Satisfaction in Exercise Scale (PNSE), the Physical Activity Self-Efficacy scale, and the Sport Commitment scale were applied. The most relevant results have showed significant differences in favor of the male gender in terms of levels of controlled motivation and amotivation, as well as higher levels of self-efficacy and basic psychological need of autonomy. Furthermore, the regression analysis has revealed that self-efficacy and current commitment to sports practice were explained by a variance of 57 and 64%, respectively, due to autonomous motivation and the basic psychological need of competence. Therefore, the basic psychological need of competence should be fostered in order to increase the levels of self-determined motivation, self-efficacy, and commitment to sports practice of the population.

## Introduction

Due to the global pandemic declared by the World Health Organization (WHO) (2020) caused by the Covid-19 virus, many changes have taken place affecting work, personal, and psychological factors, and as a result, the way people live. In Spain, on March 14, 2020, a state of alarm was decreed, which meant a period of confinement at home. In general terms (depending on the Autonomous Community), this confinement has lasted approximately 3 months. This has meant changing the daily routine, overnight, both in the way of working, which in most cases has shifted to teleworking, and in how we live our daily lives, influencing healthy lifestyles such as eating, drug consumption, rest habits and, particularly, the practice of physical activity.

The many benefits of regular physical activity for adults are well known. The World Health Organization (WHO) (2019) recommends at least 150 min of moderate activity or 70 min of vigorous activity per week for this practice to provide health benefits. Some of the most important benefits at the physical level are the improvement of body composition, image, metabolic level, and cardio respiratory capacity, helping to prevent diseases such as morbidity, sarcopenia, hypertension, and even cancer (among others; González-Carcelén et al., 2018; Jodra et al., 2019). It also produces a positive psychological effect by reducing the rate of illness due to anxiety and depression (Chan et al., 2019).

The confinement produced by Covid-19 has had a major negative psychological impact on most people (Cheval et al., 2020; Li et al., 2020). This impact has also affected people who were physically active before the confinement and have reduced their possibilities of carrying out their usual sports practice, sometimes adapting such practice at home, and in the worst case, abandoning the practice until the de-escalation began (Ammar et al., 2020; Tison et al., 2020; Woods et al., 2020), with the consequent damage to the individual’s immune system (Granados and Cuéllar, 2018).

Motivation toward sports practice is a fundamental factor for continuity and adherence (Batista et al., 2020). One of the theories that help explain motivational processes is the self-determination theory (SDT; Deci and Ryan, 1980, 1985, 1991, 2012; Ryan and Deci, 2020). The SDT determines that motivation lies within a continuum in which three levels are distinguished (Vansteenkiste et al., 2006, 2010): autonomous motivation, which includes, from most to least: self-determined intrinsic motivation, integrated regulation, and identified regulation (performing an activity for one’s own pleasure that involves practicing it); controlled motivation, which includes introjected and external regulation (these are determined by external rewards or recognition), and amotivation (least self-determined, lack of intention to practice; Deci and Ryan, 2000).

Deci and Ryan (2000) establish that this theory is based on the fact that human behavior is motivated by three basic psychological needs (BPN), which are autonomy, reflecting the desire to have the ability to choose activities, and the origin of the behavior itself (Deci, 1975; Deci and Ryan, 1985); competence, which implies a desire to produce desired results in practice (Deci, 1975; Deci and Ryan, 1985); and relationship with others, which refers to the effort to relate to and care for others, as well as to feel that others have a good relationship with you (Ryan and Deci, 2000; Deci and Ryan, 2002).

The more these BPNs are satisfied, the more self-determined the person will be toward physical activity (Hope et al., 2019). In addition, this self-determined motivation can also trigger other positive psychological aspects, such as higher levels of self-efficacy (Duchatelet and Donche, 2019) and increased commitment to sports practice (Murillo et al., 2018).

A person’s perception of self-efficacy refers to the perceived ability to overcome their fears, trust their possibilities, and face adverse situations in a positive and decisive way, decisively influencing how they think, feel, and act (Bandura, 1990; Diego-García and Zubiaur-González, 2019), while the commitment to sports practice (current and future commitment) represents the desire, the need, and the decision to continue practicing sports (Scanlan et al., 2003). This develops as we grow, relating to the perseverance and perception that one possesses in relation to one’ s capacity within the sport performed (Hernández and Capella, 2014).

A high perception of self-efficacy is important during situations of confinement and even currently, with progressive de-escalation, in relation to the continuity or not of sports practice, since, although several studies have demonstrated that the perception of self-efficacy is not a predictor of the practice of physical activity, it is an important factor (Alert et al., 2019; Tang et al., 2019).

In addition to the perception of self-efficacy, it is important to be committed to the sports practice, since the commitment that the person acquires, provided that it is by their own will, will lead them to maintain practice for a longer period of time (Podlog et al., 2015). Motivation is a key concept for the achievement of these two psychological aspects that are essential for the continuity of practice (Murillo et al., 2018; Duchatelet and Donche, 2019); however, how can motivation be maintained during a situation of confinement?

Recent studies of Covid-19 and its influence on the practice of physical activity (Hall et al., 2020; Hammami et al., 2020) have determined that many social agents (official agencies, fitness centers, personal trainers, etc.) have implemented mechanisms to facilitate the practice of physical activity from home, through written guidelines and/or online videos, for example, the recommendations of the American College of Sports Medicine (2020). However, many people have also decreased or altogether ceased their physical activity during confinement (Maugeri et al., 2020) due to a number of factors such as type of housing, means, and lack of motivation. Thus, it is necessary to understand how these psychological variables have been affected in people who practice physical activity during confinement caused by Covid-19, and how these variables are related, in order to be able to establish strategies aimed at continuity of practice.

Therefore, the aims of this study were: (a) to determine the levels of motivation, satisfaction of basic psychological needs, perception of self-efficacy, and commitment to sport of people practicing physical activity, during confinement and progressive de-escalation; (b) to determine whether there were differences between men and women in relation to these variables, to establish personalized strategies according to gender, in case these were necessary; and (c) to determine which motivational variables (levels of self-determined motivation and BPN) predict the perception of self-efficacy and commitment toward sports practice, in order to propose strategies to increase the levels of these variables and, therefore, to extend the continuity of sports practice affected by the confinement.

## Materials and Methods

The study received the approval of the Research Ethics Committee of the Rey Juan Carlos University (Madrid, Spain) following the guidelines of the Helsinki Declaration. All participants were treated in agreement with the ethical guidelines of the American Psychological Association in relation to participant consent, parent/guardian consent, confidentiality, and anonymity. Moreover, informed written consent was obtained from the participants and their parents/guardians.

### Research Design

An empirical, quantitative study was performed, using a descriptive population-based study based on surveys (Montero and León, 2007).

### Participants

The study sample was comprised of 179 Spanish subjects (90 men and 89 women) between the ages of 18 and 65 years old (*M* = 28.64; *SD* = 10.28), who practiced regular physical activity. The selection criteria were that they performed physical activity at least three times a week and 150 min of moderate/vigorous physical activity, before lockdown. Thirty-one percent were studying, 49% were working, 16% were studying and working, and 4% were retired. Sixty-three percent admitted to having lost physical form during confinement compared to 37% who did not lose form. The exclusion criteria were not answering most of the questions and unusual response patterns, although no participants were excluded. Intentional sampling was used for sample selection (Montero and León, 2007).

### Instruments

Below, the variables used in the study are listed, together with the measurement tools used:

Motivation level: The Behavioral Regulation in Sport Questionnaire (BRSQ) by Lonsdale et al. (2008) was used and validated into Spanish by Moreno-Murcia et al. (2011). This scale is composed of 36 items, which are divided into eight factors, and introduced with the phrase “I participate in this sport…”: intrinsic motivation toward knowledge (e.g., “Because I like to learn how to use new techniques”), intrinsic motivation toward execution (e.g., “Because I enjoy trying to achieve long-term goals”), intrinsic motivation toward stimulation (e.g., “Because I like to learn how to use new techniques” and “Because of the positive feelings I feel while practicing this sport”), integrated regulation (e.g., “Because it allows me to live according to my values”), identified (e.g., “Because it teaches me discipline”), introjected (e.g., “Because I would feel I had failed if I abandoned it”), external (e.g., “Because if I do not do it others would be unhappy with me”), and amotivation (e.g., “However, I wonder why I strive for this”). For this study, after the factor analysis was performed, the grouping was formed according to: autonomous motivation (intrinsic motivation toward knowledge, toward execution, toward stimulation, integrated regulation, and identified regulation), controlled motivation (introjected regulation and external regulation), and amotivation (Vansteenkiste et al., 2006, 2010).
Satisfaction of basic psychological needs: The Psychological Need Satisfaction in Exercise Scale (PNSE) was used, by Wilson et al. (2006), and validated into Spanish by Moreno-Murcia et al. (2011). This scale is composed of 18 items, which are divided into three factors and introduced with the phrase “In my training….” These factors are autonomy, composed of six items (e.g., “I think I can choose the exercises in which I participate”), competence, composed of six items (e.g., “I feel capable of completing the most challenging exercises”), and relatedness, composed of six items (e.g., “I think I get along well with my partners when we do exercises together”).
Self-efficacy: The Self-efficacy Scale of Bandura (2006) was used. This scale consists of 18 items that correspond to a single factor, self-efficacy (e.g., “When I do not feel physically well while training”). The questionnaire begins with the phrase “I am able to regularly sustain the training routine….”
Commitment to sports practice: The Sports Commitment Grade Scale of Orlick (2004) was used and validated into Spanish by Belando et al. (2012). This scale is composed of 11 items, which are divided into two factors with the opening phrase “In my trainings…”: current commitment, composed of seven items [e.g., “I have made the determination not to quit even if obstacles appear (defeats, injuries, suspensions, etc.)”], and future commitment, composed of four items (e.g., “I put 100% of my concentration and effort into the trainings, whether or not they go well”).

In all of the questionnaires, answers were provided for all of the items based on a Likert Scale of 5 points, ranging from 1, which means complete disagreement, to 5, completely agree. To facilitate the reliability of the responses by the participants, the response range of the Bandura Self-Efficacy Scale was adapted to the rest of the scales.

### Procedure

First of all, a document was created to link the four questionnaires. Following the study by Astorgano-Diez et al. (2017), the age of the participants, sex, and work situation were also collected. The Google Form platform was used, so that the questionnaire could be accessed online. The questionnaire was available on the online platform for 2 months (from May to June of 2020). The questionnaire was disseminated through different channels (WhatsApp, Facebook, Twitter, and email). The duration of the application of the questionnaire was approximately 15 min.

### Data Analysis

After performing the Kolmogorov-Smirnov normality and variance homogeneity test by means of the Levene test, it should be noted that the results obtained from both tests show a normal distribution of the data, and therefore, parametric statistics were applied.

A descriptive analysis was carried out of all the measured variables. For the tests of univariate normality, the indicators of skewness and kurtosis of variables were initially used. Curran et al. (1996) establish the limits of asymmetry and kurtosis in absolute values. Values of up to 2 for skewness and 7 for kurtosis are considered normal; values between 2 and 3 for skewness and between 7 and 21 for kurtosis are considered moderately normal; and values above 7 in skewness and 21 in kurtosis are considered non-normal. For the analysis of reliability, two indices were used, Cronbach’s Alpha (*α*; equal to or greater than 0.70; Nunnally, 1978) and the Omega Coefficient (*ω*; McDonald, 1999), which also serves to check the internal consistency of the variables used in the research and, which, according to some authors (Revelle and Zinbarg, 2009), have shown evidence of greater accuracy. This means that in McDonald’s Omega Coefficient, the established range is between 0 and 1, with the highest values giving us the most reliable measurements (Revelle and Zinbarg, 2009). With the Omega Coefficient by McDonald, the calculations were made with the “psych” 1.4.2.3 (Mullan et al., 1997) of R 3.0.3 (R Core-Team, 2014). However, according to Campo-Arias and Oviedo (2008), to consider an acceptable reliability value *via* the Omega Coefficient, this should be greater than 0.70.

A gender-based ANOVA analysis was performed, and the effect size was calculated using the following formula: Cohen’s *d* = *M*_1_ − *M*_2_/*SD*, where *SD* = √[∑ (*X* − *M*^2^)/*N*], where *X* is the raw score, *M* is the mean, and *N* is the number of cases. Following Cohen’s (1988) considerations, the effect size is considered small when the value is below 0.20, medium when it is between 0.20 and 0.50, and large when it is above 0.80. Subsequently, a stepwise regression analysis was performed.

For the analysis of the data obtained, the SPSS 23.0 statistical program was used.

## Results

### Descriptive and Reliability Analysis

The mean and SD of the measured variables were determined. The measures of skewness and kurtosis verify the univariate normality. The reliability index by means of the Cronbach’s Alpha Index and McDonald’s Omega Coefficient (see Table 1). All factors presented an adequate reliability index both for Cronbach’s Alpha (>0.70; Nunnally, 1978) and the Omega Coefficient (>0.70; Campo-Arias and Oviedo, 2008). The highest mean value was obtained for the variable autonomous motivation to practice physical activity, whereas the lowest value was obtained for the variable amotivation to practice physical activity.

**Table 1:** Descriptive and reliability analysis.

Variables	Range	*M*^1^	*SD*^2^	Skewness	Kurtosis	*α*^3^	*ω*^4^
**BRSQ**							
Autonomous motivation	1–5	4.09	0.84	−0.92	0.42	0.96	0.97
Controlled motivation	1–5	1.99	0.84	1.01	1.03	0.85	0.88
Amotivation	1–5	1.60	0.88	1.76	3.03	0.84	0.84
**BPNES**							
BPN autonomy	1–5	3.73	0.94	−0.51	−0.44	0.86	0.86
BPN competence	1–5	3.96	0.93	−0.92	0.57	0.94	0.91
BPN relatedness	1–5	3.45	0.93	−0.54	−0.19	0.82	0.87
**Self-efficacy**							
Self-efficacy	1–5	3.44	0.76	−0.17	−0.28	0.93	0.71
**Sports commitment**							
Current commitment	1–5	3.68	0.78	−0.56	−0.27	0.82	0.86
Future commitment	1–5	3.20	0.87	0.08	−0.78	0.71	0.82

1 *M*, media.

2 *SD*, standard deviation.

3 *α*, Cronbach’s Alpha.

4 *ω*, Omega Coefficient.

### Differential Analysis

The most relevant results revealed significant differences in favor of the male gender in terms of levels of controlled motivation and amotivation, as well as higher levels of self-efficacy and the basic psychological need for competence. In relation to effect size, following Cohen’s (1988) considerations, the effect size is considered small when the value is below 0.20, medium when it is between 0.20 and 0.50, and large when it is above 0.80 (see Table 2).

**Table 2:** ANOVA analysis by gender.

Variables	Female	Male	*p*^1^	Root mean square	*F*	Effect size
Autonomous motivation	3.98 ± 0.93	4.19 ± 0.73	0.09	1.98	2.79	0.25
Controlled motivation	1.84 ± 0.77	2.13 ± 0.89	0.02	3.81	5.42	0.34
Amotivation	1.46 ± 0.81	1.74 ± 0.94	0.03	3.60	4.65	0.31
BPN autonomy	3.57 ± 1.00	3.90 ± 0.86	0.02	4.72	5.39	0.35
BPN competence	3.87 ± 1.04	4.05 ± 0.79	0.18	1.52	1.76	0.19
BPN relatedness	3.36 ± 0.96	3.54 ± 0.90	0.21	1.40	1.60	0.19
Self-efficacy	3.31 ± 0.76	3.57 ± 0.75	0.02	3.03	5.24	0.34
Current commitment	3.61 ± 0.83	3.76 ± 0.72	0.20	0.99	1.62	0.19
Future commitment	3.12 ± 0.86	3.28 ± 0.88	0.22	1.14	1.49	0.18

1 *p* = significance (*p* < 0.05; *p* < 0.01).

### Linear Stepwise Regression

In order to predict the perception of self-efficacy and the current and future commitment to sport practice of the sample subjects, these variables were considered as dependent variables in their respective analyses, whereas the following were considered predictive or independent motivational variables of the tad: autonomous motivation, controlled motivation, amotivation, autonomy BPN, competence BPN, and relatedness BPN.

Linear regression analysis (successive steps) revealed that self-efficacy and current commitment to sport practice were explained by a variance of 54 and 56% for autonomous motivation and 57 and 64% for the basic psychological need for competence, respectively (see Table 3). However, future commitment to sport was not explained by any variable.

**Table 3:** Coefficients of the analysis of linear regression by successive steps.

Variables	*β*	*R^2*	*t*	*p*
**Self-efficacy**				
Autonomous motivation	0.56	0.54	7.37	0.00^1^
BPN competence	0.23	0.57	3.07	0.00
**Current commitment**				
Autonomous motivation	0.42	0.56	6.09	0.00
BPN competence	0.43	0.64	6.34	0.00

1 *p* < 0.001.

## Discussion

The aims of this study were: (a) to determine the levels of motivation, satisfaction of BPN, perception of self-efficacy, and commitment to sport practice of people who practice physical activity, during confinement and progressive de-escalation; (b) to determine if there were differences between men and women in relation to these variables; and (c) to find which motivational variables predict the perception of self-efficacy and commitment to sport practice.

Given the importance of maintaining an active style during periods of confinement, it is crucial to know some of the psychological variables that determine these processes in order to improve the quality of life of the subjects. We will now discuss the data found according to the previously formulated objectives.

In relation to the first aim, the participants of the study showed high levels of autonomous motivation toward the sports practice, which seems logical since the selected sample practiced physical activity on a regular basis. Studies such as those by Belando (2013) and Leyton-Román et al. (2020) showed that those who are more intrinsically motivated to practice physical activity are more likely to remain physically active. This is linked to the fact that the majority of participants showed high levels of satisfaction with their BPNs, slightly higher in the case of competence BPNs. According to Ryan and Deci (2020), satisfaction of the three BPNs is fundamental to achieving high levels of self-determined motivation, which makes us reflect on the importance of being able to perform the exercises and activities correctly and effectively, being able to choose which types of exercises one wants to do, and having a good relationship with others when practicing sports. In relation to self-efficacy and sports commitment, both showed high means. As mentioned, autonomous motivation greatly affects the achievement of these two variables (Murillo et al., 2018; Duchatelet and Donche, 2019), which suggests that those who practiced physical activity prior to confinement already showed high levels of autonomous motivation, self-efficacy, and current commitment to the practice of physical activity. Consequently, most continued practicing physical activity during the confinement period.

The third aim described the differences between genders in the variables studied. The most relevant results reveal significant differences in favor of men, in the levels of controlled motivation and amotivation, as well as in the BPN of autonomy and in the perception of self-efficacy. A study by López-Bueno et al. (2020) in a Spanish population determined that overall, the practice of physical activity decreased during confinement, and men decreased their activity the most. Controlled motivation and amotivation are the least self-determined forms of motivation that tend to trigger the abandonment of physical activity practice. However, men were those who obtained greater levels of satisfaction of the BPN of autonomy, which, according to studies such as those of Pérez-González et al. (2019), is related to greater levels of more self-determined motivation, with no such results found in the present study. The same occurred with the perception of self-efficacy, which was greater in men than in women, in line with the study by Chen et al. (2019) conducted in a sample of high school students, who found that the perception of self-efficacy was related to physical activity, and that these relationships were stronger in boys than in girls. Also, the power of this relationship in terms of gender was found to vary according to the age of the individuals, and, therefore, no conclusive relationships can be drawn. It is necessary to continue investigating these variables with different age groups and in situations of confinement (Chen et al., 2019).

In relation to the fourth aim, our findings revealed that self-motivation and competency BPN significantly predicted both the perception of self-efficacy and current commitment to sports practice. Many studies have established a close relationship between the BPN of competence and autonomous motivation both in the field of sports and physical activity (Prieto and Huertas-Delgado, 2019), in the field of Physical Education (Cuevas et al., 2018), and in the practice of physical activity in adults and seniors (Leyton et al., 2017). Thus, in line with the results of this study, people who are intrinsically more motivated to practice sports are those who have a greater perception of self-efficacy, as found in the studies by Neace et al. (2020) in a group of yoga practitioners, and Knight (2020) in young athletes. This prediction also occurs between more self-determined motivation and commitment to sports practice, as in the model of structural equations proposed by Pulido et al. (2018) among young soccer players.

The positive consequences related to the satisfaction of the competence BPNs originates greater levels of self-determination and consequently adaptive patterns for the sports practice. Therefore, it is necessary for public organizations and agents dedicated to sports training to use strategies aimed at improving this future commitment to practice. To this end, and according to the results obtained, the strategies to be used should focus on the satisfaction of the BPNs, with special attention to the competence BPN, since the satisfaction of the latter will improve the most self-determined motivation, and with this, the continuity and commitment to the practice. These strategies should aim to offer different exercises with the same objective so that the person can choose the exercise according to their preference or level of difficulty, set realistic and individualized activities and objectives so that the person feels capable of performing them and/or does not feel bored if they are too easy, and provide positive feedback, as well as encourage group activities and positive relatedness among participants.

It is very important to pay attention to these strategies so that, within this current situation of uncertainty, people do not stop practicing physical activity, in an effort to maintain healthy lifestyles (Dwyer et al., 2020). Identifying the level of motivation of individuals and determining the influence of the context associated with their behaviors and choices can improve interventions aimed at changing the perception of self-efficacy and commitment to sports practice, as suggested by the results of studies using SDT in relation to health (Silva et al., 2015).

Although the study presents interesting and useful results to understand how sport professionals should guide the practice of physical exercise, some limitations were found, such as the sample size. Therefore, it is necessary to carry out similar studies with larger samples, which can draw more accurate and extrapolated conclusions. It would also be interesting to replicate the study in different countries to determine differences between countries and to verify whether these psychological variables act in a similar way. It would also be very interesting to carry out a pre‐ and post-confinement measurement to see if the results have changed according to the situation.

It would be necessary to propose physical activity programs that last over time, paying special attention to the use of strategies aimed at improving the satisfaction of the competence BPN, and, therefore, increasing the forms of more self-determined motivation toward sports practice, to ensure that people increase their perception of self-efficacy, dealing more effectively with situations of risk and uncertainty, such as the present, and to maintain and/or increase the commitment to sports practice. In conclusion, the satisfaction of the BPN of competence and the autonomous motivation significantly predict the perception of self-efficacy and the current commitment to the practice of physical activity.

## Data Availability Statement

The raw data supporting the conclusions of this article will be made available by the authors, without undue reservation.

## Ethics Statement

The studies involving human participants were reviewed and approved by Comité Ético de la Universidad Rey Juan Carlos de Madrid. The patients/participants provided their written informed consent to participate in this study.

## Author Contributions

ML-R and RJ-C: conceptualization, software, validation, investigation, and resources. ML-R, RV, and RJ-C: methodology, formal analysis, and data curation. ML-R: writing – original draft preparation, writing – review and editing, and visualization. RJ-C: supervision and project administration. All authors contributed to the article and approved the submitted version.

### Conflict of Interest

The authors declare that this research was conducted in the absence of any commercial or financial relationships that could be construed as a potential conflict of interest.

## References

[ref1] Alert M. D. Saab P. G. Llabre M. M. McCalla J. R. (2019). Are self-efficacy and weight perception associated with physical activity and sedentary behavior in Hispanic adolescents? Health Educ. Behav. 46, 53–62. 10.1177/1090198118788599, PMID: 30117340

[ref2] American College of Sports Medicine (2020). Staying active during the coronavirus pandemic. Available at: https://www.exerciseismedicine.org/assets/page_documents/EIM_Rx%20for%20Health_%20Staying%20Active%20During%20Coronavirus%20Pandemic.pdf (Accessed September 16, 2020).

[ref3] Ammar A. Brach M. Trabelsi K. Chtourou H. Boukhris O. Masmoudi L.. (2020). On behalf of the ECLB-COVID19 consortium. Effects of COVID-19 home confinement on eating behaviour and physical activity: results of the ECLB-COVID19 international online survey. Nutrients 12:1583. 10.3390/nu12061583, PMID: 32481594 PMC7352706

[ref4] Astorgano-Diez A. Santos-Concejero J. Calleja-González J. (2017). Años de experiencia como factor limitante en corredores veteranos de largas distancias. Rev. Int. Med. Cienc. Act. Fis. Deporte 17, 619–632. 10.15366/rimcafd2017.68.003

[ref5] Bandura A. (1990). Perceived self-efficacy in the exercise of personal agency. J. Appl. Sport Psychol. 2, 128–163. 10.1080/10413209008406426

[ref6] Bandura A. (2006). “Guide for constructing self-efficacy scales” in Self-efficacy beliefs of adolescents. Vol. 5 eds. Pajares F. Urdan T. C. (Greenwich, CT: Information Age Publishing), 307–337.

[ref7] Batista M. Leyton-Román M. Honório S. Santos J. Jiménez-Castuera R. (2020). Validation of the Portuguese version of the Healthy Lifestyle Questionnaire. Int. J. Environ. Res. Public Health 17:1458. 10.3390/ijerph17041458, PMID: 32102461 PMC7068307

[ref8] Belando N. (2013). Motivación autodeterminada y compromiso deportivo en estudiantes adolescentes. Tesis Doctoral. Universidad Miguel Hernández de Elche.

[ref9] Belando N. Ferriz-Morell R. Moreno-Murcia J. A. (2012). Validación de la escala de grado de compromiso deportivo en el contexto español. Eur. J. Hum. Mov. 28, 111–124.

[ref10] Campo-Arias A. Oviedo H. C. (2008). Psychometric properties of a scale: internal consistency. Rev. Salud Publ. 10, 831–839. 10.1590/S0124-00642008000500015, PMID: 19360231

[ref11] Chan J. S. Liu G. Liang D. Deng K. Wu J. Yan J. H. (2019). Special issue-therapeutic benefits of physical activity for mood: a systematic review on the effects of exercise intensity, duration, and modality. J. Psychol. 153, 102–125. 10.1080/00223980.2018.1470487, PMID: 30321106

[ref12] Chen H. Dai J. Gao Y. (2019). Measurement invariance and latent mean differences of the Chinese version physical activity self-efficacy scale across gender and education levels. J. Sport Health Sci. 8, 46–54. 10.1016/j.jshs.2017.01.004, PMID: 30719383 PMC6349578

[ref13] Cheval B. Sivaramakrishnan H. Maltagliati S. Fessler L. Forestier C. Sarrazin P.. (2020). Relationships between changes in self-reported physical activity, sedentary behaviour and health during the coronavirus (COVID-19) pandemic in France and Switzerland. J. Sports Sci. 1–6. 10.1080/02640414.2020.1841396, PMID: [Epub ahead of print] 33118469

[ref14] Cohen J. (1988). Statistical power analysis for the behavioral sciences. 2nd Edn. New York: Academic Press.

[ref15] Cuevas R. García-Calvo T. González J. Fernández-Bustos J. (2018). Necesidades psicológicas básicas, motivación y compromiso en educación física. Rev. Psicol. Deporte 27, 97–104.

[ref16] Curran P. J. West S. G. Finch J. F. (1996). The robustness of test statistics to no normality and specification error in confirmatory factor analysis. Psychol. Methods 1, 16–29. 10.1037//1082-989X.1.1.16

[ref17] Deci E. L. (1975). Intrinsic motivation. New York: Plenum Press.

[ref18] Deci E. L. Ryan R. M. (1980). “The empirical exploration of intrinsic motivational processes” in Advances in experimental social psychology. ed. Berkowitz L. (New York: Academic Press), 39–80.

[ref19] Deci E. L. Ryan R. M. (1985). Intrinsic motivation and self-determination in human behaviour. New York: Plenum.

[ref20] Deci E. L. Ryan R. M. (1991). “A motivational approach to self: integration in personality” in Nebraska symposium on motivation: Perspectives on motivation. ed. Dienstbier R. (Lincoln, NE: University of Nebraska Press), 237–288. 2130258

[ref21] Deci E. L. Ryan R. M. (2000). The “what” and “why” of goal pursuits: human needs and the self-determination of behavior. Psychol. Inq. 11, 227–268. 10.1207/S15327965PLI1104_01

[ref22] Deci E. L. Ryan R. M. (2002). Handbook of self-determination research. Rochester: The University of Rochester Press.

[ref23] Deci E. L. Ryan R. M. (2012). “Self-determination theory” in Handbook of theories social psychology. eds. Kruglanski A. W. Van Lange P. A. M. Higgins E. T. (London: SAGE), 416–437.

[ref24] Diego-García M. Zubiaur-González M. (2019). Análisis de la percepción de autoeficacia en pilotos de parapente. Rev. Psicol. Deporte 28, 41–48.

[ref25] Duchatelet D. Donche V. (2019). Fostering self-efficacy and self-regulation in higher education: a matter of autonomy support or academic motivation? High. Educ. Res. Dev. 38, 733–747. 10.1080/07294360.2019.1581143

[ref26] Dwyer M. J. Pasini M. De Dominicis S. Righi E. (2020). Physical activity: benefits and challenges during the COVID-19 pandemic. Scand. J. Med. Sci. Sports 30:1291. 10.1111/sms.13710, PMID: 32542719 PMC7323175

[ref27] González-Carcelén C. M. López-Sánchez G. F. Sánchez-García C. Ibáñez-Ortega E. J. Díaz-Suárez A. (2018). Composición corporal e imagen corporal de estudiantes de Ciencias del Deporte. Sportis 4, 411–425. 10.17979/sportis.2018.4.3.3443

[ref28] Granados S. H. B. Cuéllar Á. M. U. (2018). Influencia del deporte y la actividad física en el estado de salud físico y mental: una revisión bibliográfica. Katharsis: Rev. de Cienc. Soc. 25, 141–160. 10.25057/issn.2500-5731

[ref29] Hall G. Laddu D. R. Phillips S. A. Lavie C. J. Arena R. (2020). A tale of two pandemics: how will COVID-19 and global trends in physical inactivity and sedentary behavior affect one another? Prog. Cardiovasc. Dis. 10.1016/j.pcad.2020.04.005, PMID: (in press). 32277997 PMC7194897

[ref30] Hammami A. Harrabi B. Mohr M. Krustrup P. (2020). Physical activity and coronavirus disease 2019 (COVID-19): specific recommendations for home-based physical training. Manag. Sport Leis. 1–6. 10.1080/23750472.2020.1757494

[ref31] Hernández B. Capella C. (2014). Identidad personal y compromiso deportivo en adolescentes nadadoras de nivel competitivo. Rev. Psicol. 23, 71–83. 10.5354/0719-0581.2014.32875

[ref32] Hope N. H. Holding A. C. Verner-Filion J. Sheldon K. M. Koestner R. (2019). The path from intrinsic aspirations to subjective well-being is mediated by changes in basic psychological need satisfaction and autonomous motivation: a large prospective test. Motiv. Emot. 43, 232–241. 10.1007/s11031-018-9733-z

[ref33] Jodra P. Maté-Muñoz J. L. Domínguez R. (2019). Percepción de salud, autoestima y autoconcepto físico en personas mayores en función de su actividad física. Rev. Psicol. Deporte 28, 127–134.

[ref34] Knight A. (2020). Using self-assessment to build self-efficacy and intrinsic motivation in athletes: a mixed methods explanatory design on female adolescent volleyball players. Qual. Rep. 25, 320–346.

[ref35] Leyton M. Batista M. Lobato S. Aspano M. I. Jiménez R. (2017). Application of two intervention programs in order to optimize motivation and to improve eating habits in adult and elderly women. J. Hum. Kinet. 59, 131–142. 10.1515/hukin-2017-0153, PMID: 29134054 PMC5680692

[ref36] Leyton-Román M. Núñez J. L. Jiménez-Castuera R. (2020). The importance of supporting student autonomy in physical education classes to improve intention to be physically active. Sustainability 12:4251. 10.3390/su12104251

[ref37] Li S. Wang Y. Xue J. Zhao N. Zhu T. (2020). The impact of COVID-19 epidemic declaration on psychological consequences: a study on active Weibo users. Int. J. Environ. Res. Public Health 17:2032. 10.3390/ijerph17062032, PMID: 32204411 PMC7143846

[ref65] Lonsdale C. Hodge K. Rose E. A. (2008). The behavioral regulation in sport questionnaire (BRSQ): instrument development and initial validity evidence. J. Sport Exerc. Psychol. 30, 323–355. 10.1123/jsep.30.3.323 18648109

[ref38] López-Bueno R. Calatayud J. Andersen L. L. Balsalobre-Fernández C. Casaña J. Casajús J. A. (2020). Immediate impact of the COVID-19 confinement on physical activity levels in Spanish adults. Sustainability 12:5708. 10.3390/su12145708

[ref39] Maugeri G. Castrogiovanni P. Battaglia G. Pippi R. D’Agata V. Palma A.. (2020). The impact of physical activity on psychological health during Covid-19 pandemic in Italy. Heliyon 6:e04315. 10.1016/j.heliyon.2020.e04315, PMID: 32613133 PMC7311901

[ref40] McDonald R. P. (1999). Test theory. A unified treatment. Mahwah, NJ: Lawrence Erlbaum Associates.

[ref41] Montero I. León O. G. (2007). A guide for naming research studies in psychology. Int. J. Clin. Health Psychol. 7, 847–862.

[ref42] Moreno-Murcia J. A. Marzo J. C. Martínez-Galindo C. Conte L. (2011). Validación de la Escala de “Satisfacción de las Necesidades Psicológicas Básicas” y del Cuestionario de la “Regulación Conductual en el Deporte” al contexto español. RICYDE. Rev. Int. de Cienc. del Deporte 26, 355–369. 10.5232/ricyde2011.02602

[ref43] Mullan E. Markland D. Ingledew D. K. (1997). A graded conceptualisation of selfdetermination in the regulation of exercise behaviour: development of a measure using confirmatory factor analytic procedures. Pers. Individ. Differ. 23, 745–752. 10.1016/S0191-8869(97)00107-4

[ref44] Murillo M. Sevil J. Abós Á. Samper J. Abarca-Sos A. García-González L. (2018). Análisis del compromiso deportivo de jóvenes waterpolistas: un estudio basado en la teoría de la autodeterminación. Rev. Iberoam. Psicol. Ejerc. Deporte 13, 111–119.

[ref45] Neace S. M. Hicks A. M. DeCaro M. S. Salmon P. G. (2020). Trait mindfulness and intrinsic exercise motivation uniquely contribute to exercise self-efficacy. J. Am. Coll. Health, 1–5. 10.1080/07448481.2020.1748041, PMID: [Epub ahead of print] 32343199

[ref46] Nunnally J. C. (1978). Psychometric theory. New York: McGraw-Hill.

[ref48] Orlick T. (2004). Entrenamiento mental: Cómo vencer en el deporte y en la vida. Barcelona: Paidotribo.

[ref49] Pérez-González A. M. Valero-Valenzuela A. Moreno-Murcia J. A. Sánchez-Alcaraz B. J. (2019). Revisión sistemática del apoyo a la autonomía en educación física. Apunts 138, 51–61. 10.5672/apunts.2014-0983.es.(2019/4).138.04

[ref50] Podlog L. Gustafsson H. Skoog T. Westine M. Wernere S. Alricssonf M. (2015). Need satisfaction, motivation, and engagement among high-performance youth athletes: a multiple mediation analysis. Int. J. Sport Exerc. Psychol. 13, 1–19. 10.1080/1612197X.2014.999346

[ref51] Prieto J. L. Huertas-Delgado F. J. (2019). Basic psychological needs, sport organization and levels of physical activity in scholars. Rev. Psicol. Deporte 28, 115–124.

[ref52] Pulido J. J. Sánchez-Oliva D. Sánchez-Miguel P. A. Amado D. García-Calvo T. (2018). Sport commitment in young soccer players: a self-determination perspective. Int. J. Sports Sci. Coach. 13, 243–252. 10.1177/1747954118755443

[ref53] R Core-Team (2014). R: A language and environment for statistical computing. Vienna, Austria. Available at: http://www.r-project.org/ (Accessed December 19, 2020).

[ref54] Revelle W. Zinbarg R. E. (2009). Coefficients alpha, omega, and the Gbl: comments on Sijtsma. Psychometrika 74, 145–154. 10.1007/s11336-008-9102-z

[ref66] Ryan R. M. Deci E. L. (2000). Self-determination theory and the facilitation on intrinsic motivation, social development, and well-being. Am. Psychol. 55, 68–78. 10.1037/0003-066X.55.1.68 11392867

[ref55] Ryan R. Deci E. (2020). Intrinsic and extrinsic motivation from a selfdetermination theory perspective: definitions, theory, practices, and future directions. Contemp. Educ. Psychol. 61:101860. 10.1016/j.cedpsych.2020.101860

[ref56] Scanlan T. Russell D. Beals K. Scanlan L. (2003). Project on elite athlete commitment (PEAK): II. A direct test and expansion of the Sport Commitment Model with elite amateur sportsmen. J. Sport Exerc. Psychol. 25, 377–401. 10.1123/jsep.25.3.377

[ref57] Silva F. B. Vaello A. P. Alias A. Moreno J. A. (2015). Predicción del motivo salud en el ejercicio físico en centros de fitness. RICYDE. Rev. Int. de Cienc. del Deporte 11, 163–172. 10.5232/ricyde2015.04005

[ref58] Tang M. Y. Smith D. M. Mc Sharry J. Hann M. French D. P. (2019). Behavior change techniques associated with changes in postintervention and maintained changes in self-efficacy for physical activity: a systematic review with meta-analysis. Ann. Behav. Med. 53, 801–815. 10.1093/abm/kay090, PMID: 30534971

[ref59] Tison G. H. Avram R. Kuhar P. Abreau S. Marcus G. M. Pletcher M. J.. (2020). Worldwide effect of COVID-19 on physical activity: a descriptive study. Ann. Intern. Med. 173, 767–770. 10.7326/M20-2665, PMID: 32598162 PMC7384265

[ref60] Vansteenkiste M. Lens W. Deci E. L. (2006). Intrinsic versus extrinsic goal contents in self-determination theory: another look at the quality of academic motivation. Educ. Psychol. 41, 19–31. 10.1207/s15326985ep4101_4

[ref61] Vansteenkiste M. Niemiec C. Soenens B. (2010). “The development of the five mini-theories of self-determination theory: an historical overview, emerging trends, and future directions” in Advances in motivation and achievement: The decade ahead. Vol. 16 eds. Urdan T. Karabenick S. (Bingley, UK: Emerald), 105–166.

[ref62] Wilson P. M. Rogers W. T. Rodgers W. M. Wild T. C. (2006). The psychological need satisfaction in exercise scale. J. Sport Exerc. Psychol. 28, 231–251. 10.1123/jsep.28.3.231

[ref63] Woods J. Hutchinson N. Powers S. Roberts W. Gomez-Cabrera M. C. Zsolt Radak Z. (2020). The COVID-19 pandemic and physical activity. Sports Med. Health Sci. 2, 55–64. 10.1016/j.smhs.2020.05.006 PMC7261095 34189484

[ref64] World Health Organization (WHO) (2019). Stay physically active during self-quarantine [Internet]. Dinamarca: WHO. Available at: https://www.euro.who.int/en/health-topics/health-emergencies/coronavirus-covid-19/publications-and-technical-guidance/noncommunicable-diseases/stay-physically-active-during-self-quarantine (Accessed December 19, 2020).

[ref47] World Health Organization (WHO) (2020). Opening address by the Director-General of WHO at the press conference on COVID-19 held on March 11, 2020 [Internet]. Available at: https://www.euro.who.int/en/health-topics/disease-prevention/physical-activity/news/news/2020/3/how-to-stay-physically-active-during-covid-19-self-quarantine (Accessed March 11, 2020).

